# Differential Metabolism of Medium-Chain Fatty Acids in Differentiated Human-Induced Pluripotent Stem Cell-Derived Astrocytes

**DOI:** 10.3389/fphys.2019.00657

**Published:** 2019-06-04

**Authors:** Sarah Sonnay, Anirikh Chakrabarti, Jonathan Thevenet, Andreas Wiederkehr, Nicolas Christinat, Mojgan Masoodi

**Affiliations:** ^1^ Lipid Metabolism, Nestlé Institute of Health Sciences, Lausanne, Switzerland; ^2^ Mitochondrial Function, Nestlé Institute of Health Sciences, Lausanne, Switzerland; ^3^ Institute of Clinical Chemistry, Inselspital, Bern University Hospital, University of Bern, Bern, Switzerland

**Keywords:** β-oxidation, ^13^C-metabolic flux analysis, decanoic acid, induced pluripotent stem cell-derived astrocytes, octanoic acid

## Abstract

Medium-chain triglyceride (MCT) ketogenic diets increase ketone bodies, which are believed to act as alternative energy substrates in the injured brain. Octanoic (C8:0) and decanoic (C10:0) acids, which produce ketone bodies through β-oxidation, are used as part of MCT ketogenic diets. Although the ketogenic role of MCT is well-established, it remains unclear how the network metabolism underlying β-oxidation of these medium-chain fatty acids (MCFA) differ. We aim to elucidate basal β-oxidation of these commonly used MCFA at the cellular level. Human-induced pluripotent stem cell-derived (iPSC) astrocytes were incubated with [U-^13^C]-C8:0 or [U-^13^C]-C10:0, and the fractional enrichments (FE) of the derivatives were used for metabolic flux analysis. Data indicate higher extracellular concentrations and faster secretion rates of β-hydroxybutyrate (βHB) and acetoacetate (AcAc) with C8:0 than C10:0, and an important contribution from unlabeled substrates. Flux analysis indicates opposite direction of metabolic flux between the MCFA intermediates C6:0 and C8:0, with an important contribution of unlabeled sources to the elongation in the C10:0 condition, suggesting different β-oxidation pathways. Finally, larger intracellular glutathione concentrations and secretions of 3-OH-C10:0 and C6:0 were measured in C10:0-treated astrocytes. These findings reveal MCFA-specific ketogenic properties. Our results provide insights into designing different MCT-based ketogenic diets to target specific health benefits.

## Introduction

Medium chain triglyceride-based (MCT) ketogenic diets are currently proposed as alternative brain energy substrates in conditions of limited cerebral glucose availability, such as in traumatic brain injury (TBI) (Prins and Matsumoto, 2014; Bernini et al., 2018), patients with glucose transporter 1 deficiency (Krass et al., 2016) and Alzheimer’s disease (Reger et al., 2004; Castellano et al., 2015; Cunnane et al., 2016; Croteau et al., 2018). Moreover, besides having antioxidant properties (Jarrett et al., 2008), ketogenic diets were reported to be important in seizure control in epileptic patients (Pulford, 1927; Neal et al., 2008). MCTs are glycerolesters usually carrying a mixture of octanoic (C8:0) and decanoic (C10:0) acid. Yet, the rationale behind the selection of C8:0 and C10:0 is unclear. Upon supplementation, MCT are hydrolyzed in the gut, which leads to an increase in the plasma concentrations of the medium chain fatty acids (MCFA) (i.e., C8:0 and C10:0) (Bernini et al., 2018). In the brain, MCFA cross the blood-brain barrier (Wlaź et al., 2015) and are oxidized in cells (Ebert et al., 2003) through β-oxidation for acetyl-CoA (AcCoA) generation and subsequent production of ketone bodies (Auestad et al., 1991), namely β-hydroxybutyrate (βHB) and acetoacetate (AcAc). Those produced ketone bodies can further be used as metabolic fuel by the brain (Nugent et al., 2014; Andersen et al., 2017; Evans et al., 2017).

While astrocytes have been restricted to a scaffold-associated function in the brain for decades, new lines of evidence suggest the important role of astrocytic oxidative metabolism for supporting brain function (Sonnay et al., 2016, 2017, 2018), as well as the preferential stimulation of astrocytic metabolism by fatty acids, and subsequent ketone bodies production (Edmond et al., 1987; Thevenet et al., 2016).

Based on our initial observations (Thevenet et al., 2016) and although C10:0 and C8:0 differ in length by only two carbons, we hypothesize that they differ in their ability to contribute to ketogenesis. In astrocytes, the metabolic pathways giving rise to ketone bodies remain unclear (Thevenet et al., 2016) and new insights into astrocytic ketogenesis and β-oxidation may help to define the optimal MCFA composition of the ketogenic diets. We have recently observed a significant but modest increase in plasma C8:0 and C10:0 in the subset of TBI patients after receiving high level MCT enteral nutrition (Bernini et al., 2018). Interestingly, administration of higher MCT concentrations, compared to commonly used formula, did not have significant impact on the level of ketones produced. This further confirms the importance of identifying the optimal formulation to raise brain ketones to therapeutically relevant levels.

**Figure 1:**
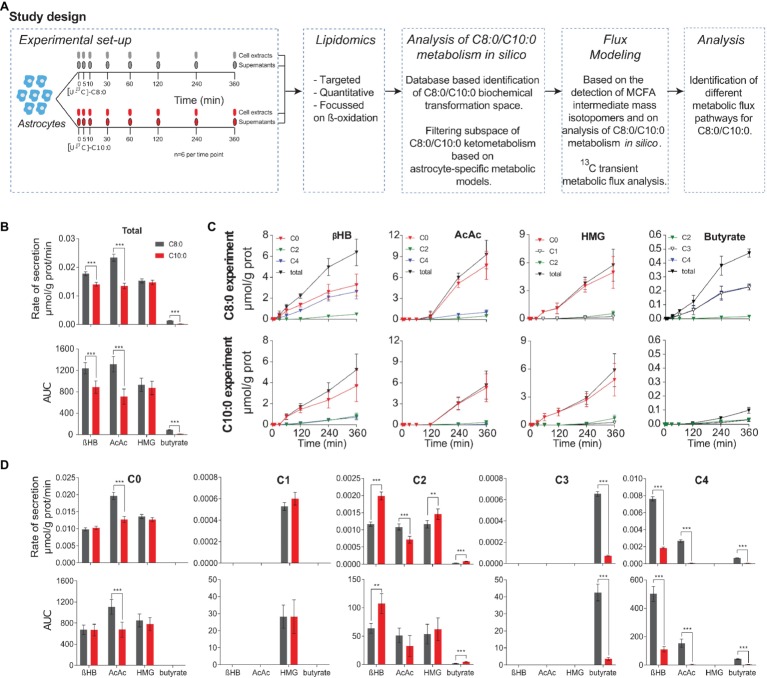
**(A)** Overview of the experimental set-up and study design. Human iPSC astrocytes were treated with [U-^13^C]-C8:0 or [U-^13^C]-C10:0. Supernatants and cell monolayers were collected at 0, 5, 30, 60, 120, 240, and 360 min, and analyzed by LC-MS. MCFA intermediates involved in β-oxidation and ketone bodies production were quantified. Analysis of C8:0 and C10:0 metabolism *in silico* was performed, and a mathematical (sub-network) based on the latter and on the detected metabolites was built to fit the experimental ^13^C isotopomer FE curves. The resulting fluxes were compared between conditions. **(B)** Secretion rates and AUC of selected ketone bodies, HMG and butyrate determined from the total extracellular concentrations experimentally measured. **(C)** Changes in mass isotopomer concentrations of ketone bodies, HMG and butyrate measured in the medium over time for the C8:0 and C10:0 experiments. **(D)** Secretion rates and AUC determined from experimentally measured extracellular concentrations for each mass isotopomer **(C)**. **p < 0.01; ***p < 0.001.

Therefore, we investigated β-oxidation of C8:0 and C10:0 to assess the underlying metabolic network, differences in metabolic flux pathways and ketogenesis at the basal level. We aimed to provide insights into the production of ketones in the brain and potential differences in metabolic properties of MCFA, namely differential secretions of MCFA intermediates and glutathione formation. Using our established cellular model (Thevenet et al., 2016), we tracked the metabolism of [U-^13^C]-C8:0 and [U-^13^C]-C10:0 in differentiated human-induced pluripotent stem cell-derived (iPSC) astrocytes by using liquid chromatography-mass spectrometry (LC-MS), conducted an integrated analysis *in silico* to identify and characterize specific reactions leading to the production of βHB, and formulated a reduced (sub-network) astrocyte-specific mathematical model of β-oxidation that was fitted to the experimental data (Figure 1A). The results indicate significant differences in MCFA metabolism. In particular, astrocytes incubated with C8:0 release βHB, AcAc and butyrate in larger amounts and at higher rates than with C10:0, with the major fraction coming from unlabeled substrates (irrespectively of the initial MCFA). Moreover, flux analysis indicates opposite direction of metabolic flux between the MCFA intermediates C8:0 and C6:0, with an important fraction of unlabeled sources contributing to the elongation steps in the case of C10:0, suggesting different β-oxidation pathways. Finally, larger intracellular glutathione concentrations, indicating potential additional antioxidant benefits, as well as higher secretions of 3-OH-C10:0 and C6:0 were measured in C10:0-treated astrocytes. Overall, these findings suggest C8:0- and C10:0-specific ketometabolic traits relevant for further studies on designing optimal ketogenic nutritional interventions using appropriate C8:0/C10:0 ratios to reach specific health benefits.

## Materials and Methods

### Chemicals and Reagents

Acetonitrile, ethanol, and isopropanol (LC-MS grade) were purchased from VWR Internationals (Leuven, Belgium) and Merck (Darmstadt, Germany). Water was purified in-house using a Milli-Q Advantage A10 system from Merck Millipore (Billerica, MA, USA). Acetic acid was supplied by Sigma-Aldrich (St. Louis, MO, USA). Chemicals used for internal and external calibration were purchased from Sigma-Aldrich (St. Louis, MO, USA), Larodan (Solna, Sweden), Toronto Research Chemical (Toronto, Canada), CDN Isotopes Inc. (Pointe-Claire, Canada), and Cambridge Isotopes Laboratories (Tewksbury, MA, USA). Uniformly labeled tracers ^13^C_8_-octanoic acid [>99% ^13^C, [U-^13^C]-C8:0] and ^13^C_10_ decanoic acid [>98% ^13^C, [U-^13^C]-C10:0] were purchased from Cambridge Isotopes Laboratories (Tewksbury, MA, USA). Stocks of [U-^13^C]-C8:0 and [U-^13^C]-C10:0 were prepared in dimethyl sulfoxide (DMSO) at 100 mM with 0.3% DMSO final concentration.

### Liquid Chromatographic Separation and Mass Spectrometric Detection

Liquid chromatography was performed on a I-Class UPLC system (Waters Corporation, Milford, MA, USA) combining a binary pump, a FTN autosampler and a column oven. Chromatographic separation was achieved on a Waters ACQUITY UPLC BEH C8 Column (100 mm × 2.1 mm, 1.7 μm) with binary solvent system at a flow rate of 450 μl/min. Mobile phase A was 0.1% acetic acid in water and B was 0.1% acetic acid in acetonitrile/isopropanol (1:1). The binary solvent gradient was as follow: 0.0–1.0 min at 0% B, 1.0–6.5 min from 0 to 100% B, 6.5–8.5 min 100% B, followed by 2 min of equilibration at initial conditions. Column oven temperature was set to 55°C and the autosampler injection volume to 1 μl.

High resolution mass spectrometric analysis was performed on a Q Exactive mass spectrometer (ThermoFisher Scientific, Bremen, Germany) operating in negative ionization mode over the mass range *m/z* 65–600 with a resolving power of 70,000 (at *m/z* = 200). Data was acquired in profile mode with an AGC target of 5e6 ions and a maximum injection time of 250 ms. The mass spectrometer was interfaced to the UPLC system using a HESI probe. The spray voltage was set to −4 kV. The heater and capillary temperatures were both set to 350°C. Sheath gas and auxiliary gas flow rate were set to 45 and 15 AU, respectively. The instrument was calibrated every 4 days according to manufacturer specifications.

### Cell Culture

All experiments were approved by the Swiss Ethics Committees on Research Involving Humans. Differentiated human iPSCs (iCell astrocytes) were obtained from Cellular Dynamics International (CDI, Madison, WI, USA). The cells were thawed according to the manufacturer’s instruction. iPSC astrocytes were cultured in DMEM containing 25 mM glucose, 1 mM pyruvate, and 4 mM glutamine (Thermo Scientific-Life Technologies, ref. 41966029) supplemented with 10% fetal calf serum and N2 complement. Cells were kept in culture in a humidified atmosphere (5% CO_2_) at 37°C for 7 days with medium changed at days 3 and 6.

### Sample Preparation

A 0.1–0.5 μM internal standard solution was prepared by mixing individual stock solutions with mobile phase A. This solution contained heavy labeled compounds, which do not interfere with measured isotopomers and was used to dilute media samples and reconstitute cell extracts.

Cells were prepared as previously described (Thevenet et al., 2016). Briefly, iPSC astrocytes were washed three times and incubated in serum- and glutamine- free DMEM A14430-01 (Thermo Scientific-Life Technologies) containing 1 mM glucose, N2 supplement, and 300 μM [U-^13^C]-C8:0 or [U-^13^C]-C10:0 for up to 6 h (1 ml/well). Experiments were run in duplicates in three independent experiments (*n* = 3). Cell supernatant was collected at different time points (0, 5, 10, 30, 60, 120, 240, and 360 min) and stored at −80°C until analysis. On analysis day, 25 μl of cell culture supernatant was mixed with 25 μl of dilution solution (50 μl internal standard stock solution diluted with 450 μl mobile phase A) in a PCR plate. The plate was sealed, placed in a Thermomixer Comfort C maintained at 4°C, and shaken for 5 min at 1,000 rpm. Samples were placed in the autosampler and immediately analyzed.

Cell monolayers were collected at different time points (0, 5, 10, 30, 60, 120, 240, and 360 min). At each time point, cells were immediately placed on ice to stop metabolism, washed with cold PBS, and frozen. After collection, cell pellets were extracted once with 500 μl and once with 700 μl ethanol/water (7:3) followed by centrifugation at 17,500 g for 20 min. Supernatants were dried under vacuum at room temperature and the solid residues were reconstituted in 30 μl of mobile phase A and immediately analyzed. The pellets were stored at −80°C until protein content quantification.

### Protein Content

Protein content was determined with a bicinchoninic acid-based protein assay (BCA protein assay kit 10678484; Thermo Scientific) with bovine serum albumin as standard.

### Biochemical Pathway Analysis

The objectives of this analysis were to identify astrocyte-specific metabolic intermediates and metabolic routes of C8:0-C10:0 to βHB transformation including key intermediates (e.g., AcCoA, AcAc and 3-Hydroxy-3-methyl glutarate (HMG)). This consisted of two steps. The first step was the enumeration of paths from compound of interest (i.e., C8:0-C10:0) and target of interest (i.e., βHB). In this regard, we used the KEGG database (Kanehisa and Goto, 2000; Kanehisa et al., 2016, 2017) and specifically, the PathComp methodology (Goto et al., 1997) to identify the human-specific metabolic paths between the compounds of interest. We limited the search of paths to a maximum length of 20 (i.e., 20 biochemical transformations from the starting compound). The second step was the refinement of the transformations identified in step one based on genome scale metabolic models (i.e., astrocyte specificity) (Schultz and Cutub, 2016). In this regard, we used the astrocyte-neuronal metabolic model published by Özcan and Çakır (2016). The model provided curated astrocyte-specific metabolic information for 276 metabolites interacting across 375 reactions. Out of the metabolites and reactions participating in the paths identified in the first step, only those implicated in the astrocyte model were considered for further analysis.

Network analysis and visualization of paths, interconnectivities, and cellular specificities were performed using custom Matlab (Mathwork Inc.) scripts and edited using yEd (yWorks GmbH).

### Modeling of Experimental ^13^C Curves

Chromatogram extraction using a mass tolerance of 5 ppm and calculation of ratios of mass isotopomers to internal standard were performed in Xcalibur Software 2.2 SP1. Absolute concentrations were calculated by multiplying the area ratio by the internal standard amount. Time-course data (cells and media) were subtracted to initial conditions (i.e., cells and media before incubation, respectively) and normalized to protein content. Similar procedure was performed in an unrelated cell line (i.e., HepG2 cells) under similar experimental conditions for astrocytic-specificity measurements validation. Key metabolites are reported in Supplementary Figure S1. Then, correction for substrate purity and natural abundance of the isotope, and calculation of fractional enrichments (FE) were performed with IsoCor Software 1.0 (Millard et al., 2012). Mass isotopomers differ by the number of ^13^C atoms in the molecule. C0 represents the fraction of unlabeled molecules in the total molecule pool, C1 the fraction of molecules labeled in one carbon, C2 in two carbons, etc. The sum of all fractions is one.

Isotopic transient ^13^C-metabolic flux analysis of [U-^13^C]-C8:0 and [U-^13^C]-C10:0 through β-oxidation was based on the intracellular and extracellular detection of the derivative metabolites. FE < 1% were not considered in the modeling process, because they were associated with large uncertainty. The metabolic model describing ^13^C labeling was solved mathematically by a set of linear differential mass isotopomer-balance equations describing the stoichiometry of the system at equilibrium and assuming negligible cellular growth (Supplementary Data). Metabolic steady-state and isotopic transient-state were considered during the experiment, as during incubation of the labeled substrate, the total intracellular pools of free acids remained unchanged and the distribution of mass isotopomers changed with time according to the flux network. The model was fitted to the experimental measurements by minimizing the residual sum of squares using least square regression. Variance of the estimated fluxes was calculated with Monte Carlo (MC) simulations using artificially generated dataset by adding Gaussian noise with similar variance of the fit residuals to the best fit. Initial values were randomly generated within confidence interval of the obtained fluxes. All numerical procedures were performed in Matlab (Mathwork Inc.).

All pool sizes were determined from the LC-MS measurements, except the pool of AcCoA, whose value was set to 0.027 μmol/mg prot (Amaral et al., 2011). As extracellular metabolite concentrations increased over time, net specific transmembrane rates were calculated experimentally from the slope of a linear regression to those changes. The resulting values were fixed in the modeling process.

### Statistics

Data are shown as mean ± SD. For experimental measurements, the SD was determined from three independent experiments, which were run in duplicate. For metabolic fluxes, the SD was determined from fitting a Gamma function to the histograms obtained from at least 500 MC. Flux comparison between C8:0 an C10:0 experiments was performed by permutation analysis with 2000 permutations followed by individual two-tailed student *t*-test. Experimental measurements comparison between C8:0 and C10:0 experiments was also performed by individual two-tailed student *t*-test. All reported *p*’s were corrected for multiple comparisons using Holm-Bonferroni method (Holm, 1979). All calculations include error propagation.

## Results

### Ketone Bodies Secretion

To determine the contribution of C8:0 and C10:0 to the release of ketone bodies, butyrate, and HMG in the medium, mass isotopomers, namely βHB (C0, C2, C4), AcAc (C0, C2, C4), HMG (C0, C1, C2), and butyrate (C2, C3, C4), were quantified over time. The release rates were calculated by linear fitting of these extracellular concentration profiles (total = labeled + unlabeled) and the areas under the curve (AUC) of the total concentrations were determined (Figure 1B). In particular, a significant (*p* < 0.001) decrease in secretion rates and AUC of total βHB, total AcAc, and total butyrate was observed in the C10:0 experiment as compared to the C8:0. Noticeably, there was a different time delay of ketone secretion between the C8:0 and C10:0 condition. In the C8:0 experiment, secretion of βHB, AcAc, and butyrate was detected at time 30, 120, and 30 min, respectively, while in the C10:0 condition, it was detected at time 60, 240, and 120 min, respectively (Figure 1C). These results indicate that both the rates of secretion and the total amounts of produced ketone bodies and butyrate are higher when astrocytes metabolize C8:0 compared to C10:0. No difference in total HMG secretion rate and AUC were measured.

Separation of the data into concentrations of mass isotopomers is shown in Figures 1C,D. In particular, in the case of AcAc a decrease (*p* < 0.001) in both AUC and secretion rates were measured in the unlabeled and fully labeled fractions of the C10:0 as compared to the C8:0 experiment. The secretion rate of AcAc C2 was also significantly lower (*p* < 0.001) in the C10:0 condition as compared to the C8:0. The secretion rates and AUC of βHB C2 and βHB C4 were also significantly different according to the MCFA under investigation. A significant decrease of βHB C4 secretion rate (*p* < 0.001) and AUC (*p* < 0.001) was detected in the C10:0 condition as compared to the C8:0. The opposite was found for βHB C2 secretion rate (*p* < 0.001) and AUC (*p* < 0.01). No significant difference was measured in the AUC of the different mass isotopomers of HMG. However, the secretion rate of HMG C2 was significantly (*p* < 0.01) higher in the C10:0 condition as compared to C8:0. Regarding butyrate, larger (*p* < 0.001) secretion rates and AUC were identified in C3 and C4 in the C8:0 condition as compared to the C10:0. Finally, the unlabeled/(unlabeled + labeled) ratio at 360 min of βHB and AcAc was 51 and 71%, 83 and 93%, for C8:0 and C10:0, respectively.

### Biochemical Pathway Analysis *in silico* of C8:0-C10:0 Metabolism

To further investigate the observed differences between the impact of C8:0 and C10:0, biochemical pathway analysis *in silico* was employed to parse the known C8:0-C10:0 biochemical transformation space to identify downstream metabolites for measurement and flux modeling.

**Figure 2:**
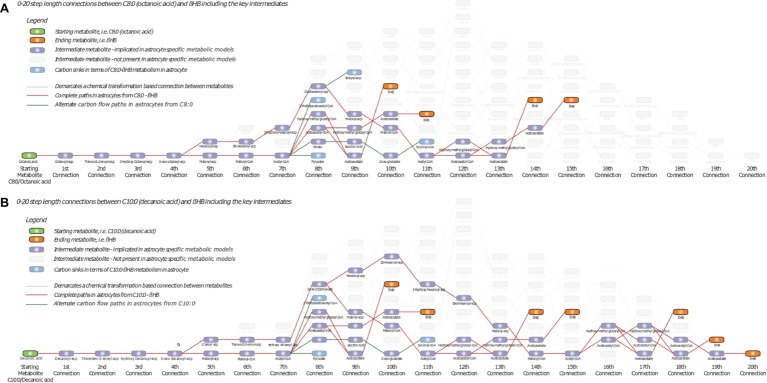
Biochemical transformation space of **(A)** C8:0-βHB and **(B)** C10:0-βHB, starting from C8:0 or C10:0 on the left (green node) to βHB on the right (orange nodes). Additional nodes in purple indicate intermediate metabolites, while lines indicate chemical transformation-based connections between metabolites. Grey nodes indicate alternate intermediates not implicated in astrocytes based on genome scale metabolic model. Red lines indicate complete paths of transformation of C8:0 or C10:0 to βHB in astrocytes. Green lines ending with blue nodes indicate incomplete paths of C8:0 or C10:0 metabolism in astrocytes leading to carbons routing away from βHB. ACP, Acyl-carrier protein.

In the case of the C8:0 to βHB transformation space, we identified 73 paths (minimum length 10, maximum length 20), involving 84 unique metabolites interconnected by 91 unique reactions (Figure 2A), considering the overall human metabolism. Similarly, in the case of the C10:0 to βHB transformation space, we identified 67 paths (minimum length 10, maximum length 20), involving 82 unique metabolites interconnected by 89 unique reactions (Figure 2B). Upon imposing astrocyte specificity (Özcan and Çakır, 2016), this transformation space was reduced to 33 metabolites and 37 reactions in the case of C8:0-βHB (Figure 2A), and 47 metabolites and 56 reactions in the case of C10:0-βHB (Figure 2B). These filtered paths were used as the biochemical transformation space for analysis of C8:0 and C10:0 metabolism, and formulation of flux models (sub-networks). On one hand, these paths showed complete paths from C8:0-C10:0 to βHB, highlighting key intermediates (e.g., AcCoA, AcAc, and HMG). On the other hand, they also highlighted astrocyte-specific metabolic intermediates of C8:0-C10:0 degradation, wherein carbon introduced as C8:0-C10:0 could accumulate without being transformed to target compound formation (e.g., βHB and butyrate) (Figures 2A,B). Note that due to database limitation in terms of bound forms, the resulting metabolites are often only documented in the acyl-carrier protein (ACP)-bound form (wherever available). The purpose of Figure 2 is to provide the global network of MCFA metabolism.

### Modeling of the Experimental ^13^C-Labeled Curves

**Figure 3:**
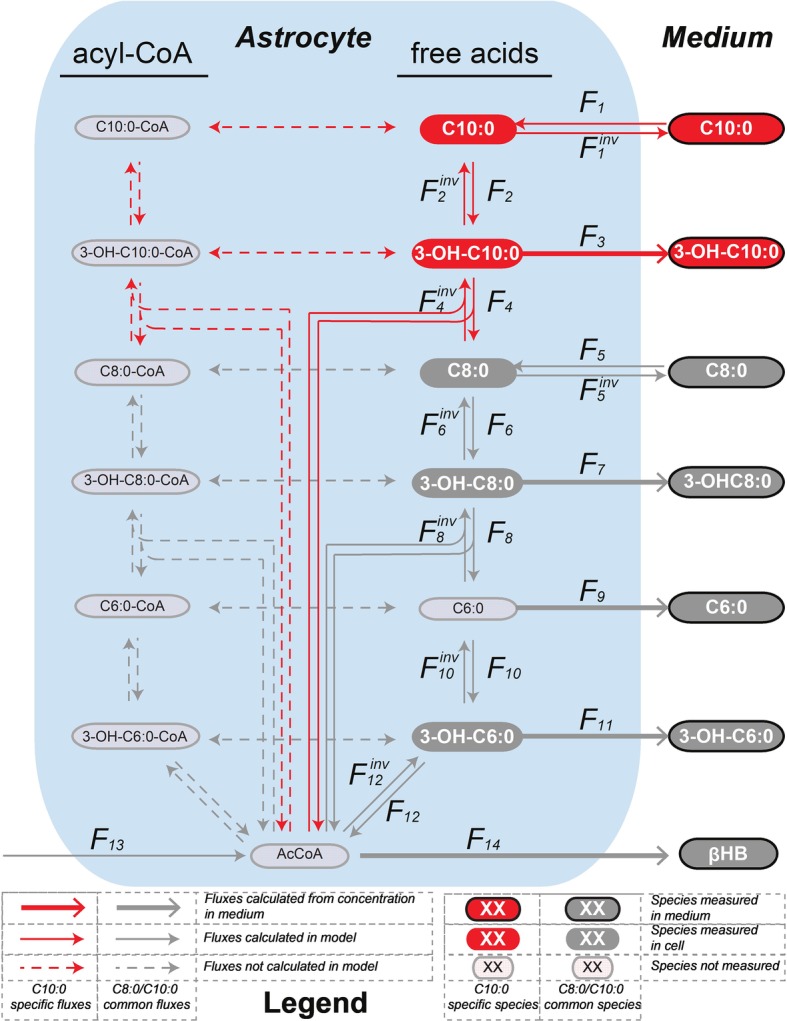
Mathematical model of β-oxidation (and elongation–reverse fluxes)—sub-network—to the production of βHB used to fit the ^13^C isotopomer FE curves. The model used to fit the C8:0 and C10:0 data is shown in grey, and in grey and red, respectively. The free acids were detected by LC-MS, while the acyl-CoA-bound forms were not. Therefore, the acyl-CoA part was considered as a proxy for the free acid part. F_3_, F_5_^inv^ (in the case of C10:0), F_7_, F_9_, F_11_ and F_14_ (in the case of C8:0 and C10:0) were fixed to the extracellular concentrations experimentally measured. F_1_, F_1_^inv^ (for C10:0) and F_5_, F_5_^inv^ (for C8:0) were let to adjust in the modeling process.

The mathematical model of β-oxidation to the production of βHB (Figure 3) used to fit the ^13^C isotopomer FE curves was based on Figure 2 and restricted to the detection of the mass isotopomers of the MCFA intermediates by LC-MS. In particular, only the fully labeled and fully unlabeled parts of the MCFA intermediates could be detected in both intracellular and extracellular space. Only unlabeled hexanoic acid (C6:0) was detected intracellularly, while both fully labeled and fully unlabeled fractions could be measured extracellularly. None of the mass isotopomer of butyrate could be detected intracellularly (presumably because the volatile butyric acid evaporated during extraction (solvent removal) under vacuum), while butyrate C2, C3, and C4 were present in the medium. The unstable AcAc was also probably degraded during cell pellet extraction and was thus undetectable intracellularly, but AcAc C0, C2, and C4 were measured in the medium. Finally, only unlabeled βHB was detected in astrocytes, while βHB C0, C2, and C4 were measured extracellularly. The network included both degradation (top-down direction) and elongation (bottom-up direction) of MCFA. Only the MCFA free acids could be detected in the LC-MS measurements, and therefore the reactions involving the acyl-CoA-bound forms were considered as a proxy for the sub-network involving the free acid forms (Figure 3). The free acid pool sizes, which remained constant over time, were measured by LC-MS, except the AcCoA that was assumed to be 0.027 μmol/mg prot (Amaral et al., 2011). In the C8:0 experiment, the initial intracellular concentrations of C8:0, 3-OH-C8:0, C6:0 and 3-OH-C6:0 were 0.100 ± 0.056 μmol/g prot, 0.008 ± 0.004 μmol/g prot, 0.060 ± 0.035 μmol/g prot, and 0.002 ± 0.001 μmol/g prot, respectively. In the C10:0 experiment, the initial intracellular concentrations of C10:0, 3-OH-C10:0, C8:0, 3-OH-C8:0, C6:0, and 3-OH-C6:0 were 0.156 ± 0.066 μmol/g prot, 0.003 ± 0.001 μmol/g prot, 0.131 ± 0.072 μmol/g prot, 0.008 ± 0.004 μmol/g prot, 0.048 ± 0.038 μmol/g prot, and 0.002 ± 0.001 μmol/g prot, respectively.

**Figure 4:**
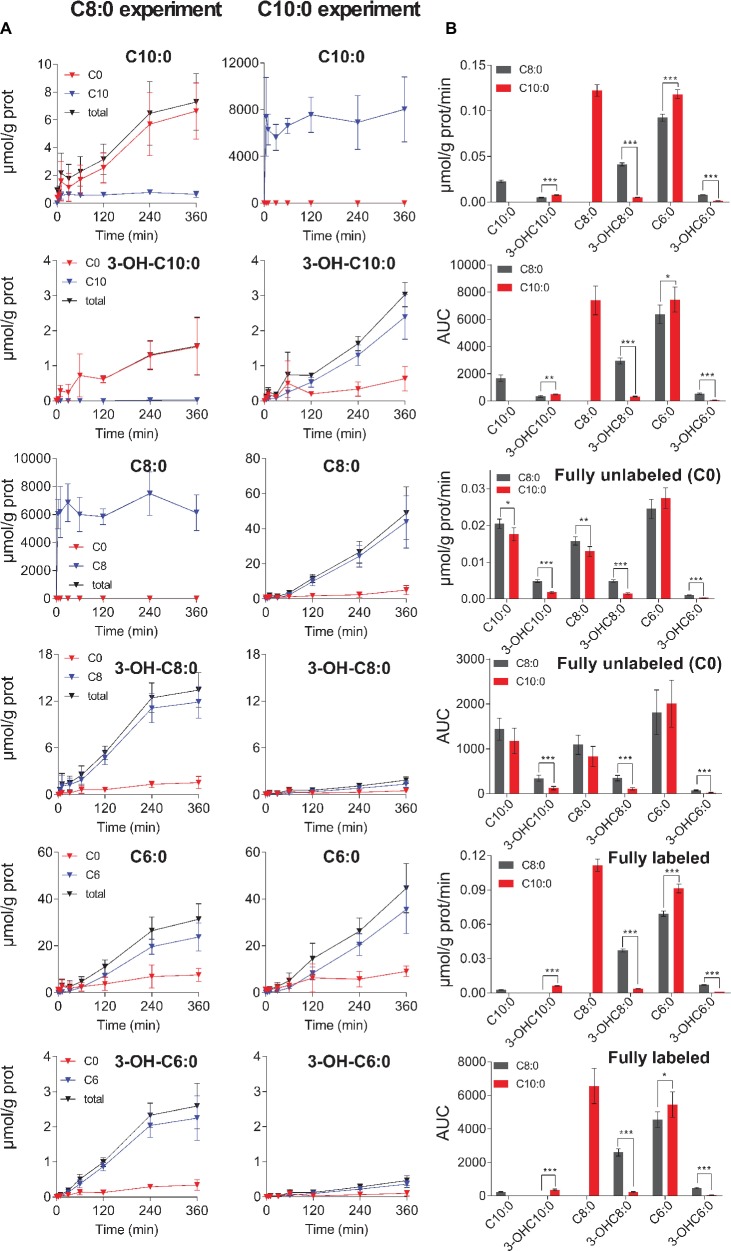
**(A)** Changes in extracellular concentration over time of unlabeled (C0), fully labeled and total (unlabeled + fully labeled) MCFA intermediates measured in the C8:0 (on the left) and in the C10:0 (on the right) experiments. **(B)** Depicts the secretion rates and the AUC of total, unlabeled and fully labeled MCFA intermediates. Fully labeled C8:0 in the C8:0 experiment and C10:0 in the C10:0 experiment are not shown, because they are the incubation substrates.

The net transmembrane rates were fixed to the values determined from the measurements of the MCFA intermediate (F_3_, F_5_^inv^ for C10:0, F_7_, F_9_, F_11_ for C8:0 and C10:0) and βHB (F_14_) total concentrations in the medium over time for each condition. Only the transmembrane exchanges (F_1_ and F_1_^inv^ for C10:0 and F_5_ and F_5_^inv^ for C8:0) at the level of the initial substrate were let to adjust and considered reversible to allow equilibration with the medium. F_2_, F_6_ and F_10_ convert the MCFA intermediates into their hydroxy-(3-OH) counterparts (F_2_^inv^, F_6_^inv^ and F_10_^inv^ are responsible for the reverse reactions). In those reactions, the carbon positions and numbers are maintained. In F_4,_ F_8_ and F_12_ reactions carbons are lost (or gained in the case of the reverse reactions) in AcCoA and the resulting molecules are two carbons (F_4_ and F_8_) or four carbon (F_12_) shorter. The network applied to the C8:0 data is shown in grey in Figure 3. It was extended (in red) to the C10:0 data. Although labeled 3-OH-C10:0 and C10:0 was detected in the medium (Figure 4), this extended model was not applied to the C8:0 data, because no labeled 3-OH-C10:0 could be detected intracellularly and FE of intracellular C10:0 was associated with large SD (FE < 1%). A dilution flux at the level of AcCoA (F_13_) was added to take into account potential utilization of unlabeled substrates present in the medium, namely glucose (Zwingmann and Leibfritz, 2003) and amino acids (i.e., isoleucine and leucine) (Bixel and Hamprecht, 1995; Dienel and Cruz, 2009).

### Fluxes of β-Oxidation to Ketone Bodies Secretion

Significantly higher (*p* < 0.001) secretion rates of total 3-OH-C10:0 (F_3_^C8^ = 0.0048 ± 0.0004 μmol/g prot/min *vs.* F_3_^C10^ = 0.0079 ± 0.0004 μmol/g prot/min) and total C6:0 (F_9_^C8^ = 0.093 ± 0.004 μmol/g prot/min *vs.* F_9_^C10^ = 0.118 ± 0.005 μmol/g prot/min) were measured in the C10:0 condition as compared to the C8:0. Higher contribution (*p* < 0.001) of fully labeled carbons were measured in both 3-OH-C10:0 and C6:0 under C10:0 as compared to C8:0. On the contrary, the contribution of unlabeled carbons in 3-OH-C10:0 was higher (*p* < 0.001) in the C8:0 experiment as compared to C10:0. Reversely, higher (*p* < 0.001) secretion rates of total 3-OH-C8:0 (F_7_^C8^ = 0.0414 ± 0.0016 μmol/g prot/min *vs.* F_7_^C10^ = 0.0051 ± 0.0003 μmol/g prot/min) and total 3-OH-C6:0 (F_11_^C8^ = 0.0079 ± 0.0003 μmol/g prot/min *vs.* F_11_^C10^ = 0.0013 ± 0.0001 μmol/g prot/min) were measured in the case of C8:0 as compared to C10:0. The contribution of both fully labeled and unlabeled carbons were higher (*p* < 0.001) in the C8:0 experiment as compared to the C10:0 (Figure 4).

**Figure 5:**
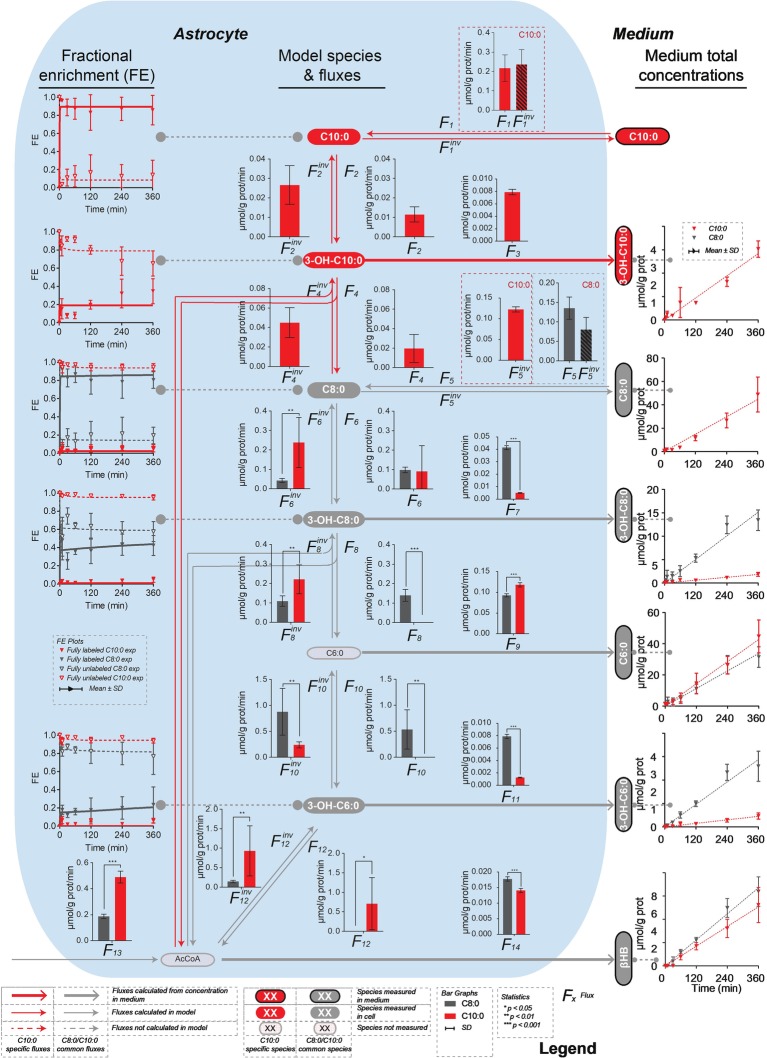
Metabolic flux pathways for C8:0 and C10:0. On the left, ^13^C isotopomer FE curves and best fit of the mathematical model. On the right, linear regression of the extracellular concentrations experimentally measured and used to fix the transmembrane fluxes. The bar graphs represent the fluxes resulting from the model best fit. Each flux is shown with its associated SD.

The model generally mimicked the measured labeling curves in both C8:0 (*R*^2^ = 0.972) and C10:0 (*R*^2^ = 0.987) conditions (Figure 5). Supplementary Table S1 summarizes the fluxes computably determined and the calculated flux differences.

### Effect of Medium-Chain Fatty Acids on Other Metabolic Pathways

**Figure 6:**
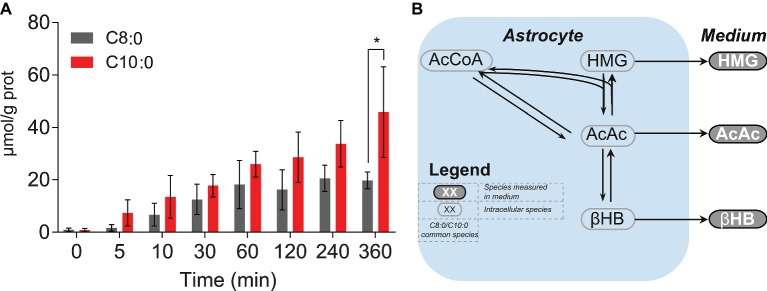
**(A)** Intracellular concentrations of unlabeled glutathione (reduced form) experimentally measured over time. **(B)** KEGG-based schematic overview of ketone bodies (i.e., AcAc, HMG and βHB) production from AcCoA.*p < 0.05.

To further investigate the antioxidant properties of C8:0 and C10:0, intracellular unlabeled concentrations of glutathione (reduced form) were measured over time. Significant (*p* < 0.05) higher concentration after 360 min was found in the C10:0 experiment as compared to the C8:0 (Figure 6A). No labeled glutathione was detected.

## Discussion

Although MCT-based ketogenic diets have received growing interest during the past few years, metabolism of C8:0 and C10:0 remains unclear. Thus, understanding the kinetics of βHB production and β-oxidation from MCFA would allow us to better define the rationale for optimal ketogenic composition to not only better provide ketones in the brain but also take advantage of other metabolic properties to reach specific health benefits. Based on the differences observed on the redox ratio produced by the oxidation of C8:0 and C10:0 (Thevenet et al., 2016), and on the fact that administration of higher level MCT did not have a significant impact on the concentration of ketone bodies in the brain of TBI patients, as compared to a standard formula (Bernini et al., 2018), we aimed at tracking the basal production of βHB from C8:0 and C10:0 at the cellular level using metabolic flux analysis with^13^C-labeled substrates. In this regard, we conducted an integrative analysis *in silico* of ketometabolism in astrocytes, as well as experimental analysis using [U-^13^C]-C8:0 and [U-^13^C]-C10:0. Overall, this study demonstrates that the metabolic pathways underlying the β-oxidation of MCFA of different lengths, namely C8:0 and C10:0, to the production of ketone bodies differ in human iPSC astrocytes. This differences stand not only in the total amount and mass isotopomers of MCFA intermediates and ketone bodies that are secreted, but also in the intracellular fluxes.

We observed that astrocytes incubated with C8:0 secreted more ketone bodies, namely βHB and AcAc, as well as butyrate, both in terms of production rates and total concentrations, compared to C10:0. Interestingly, most of the ketone bodies production arose from unlabeled sources present in the medium, suggesting that iPSC astrocytes do not exclusively require MCFA to produce βHB. This observation could in part explain the lack of increase in ketone bodies recently measured in TBI patients (Bernini et al., 2018). The larger extracellular concentration of βHB found in the case of C8:0 is in line with our previous report, in which unlabeled C8:0 stimulated ketogenesis, as compared to unlabeled C10:0 and control (Thevenet et al., 2016). Furthermore, in the same study, extracellular βHB levels were similar in C10:0 and control experiments, indicating that iPSC astrocytes have the ability to produce βHB in the absence of MCFA (Thevenet et al., 2016). In addition, more fully labeled βHB (βHB C4) was produced under C8:0, while more partially labeled βHB (βHB C2) was secreted under C10:0. As the concentration of unlabeled βHB was similar between the two conditions (Figure 1D), the larger production of βHB in the case of C8:0 came from βHB C4 (Figures 1B–D). The secretion profile of AcAc, the precursor of βHB (Figure 6B) (Kanehisa and Goto, 2000; Kanehisa et al., 2016, 2017), was different to the one of βHB. In particular, while C8:0 produced more AcAc C4 than C10:0, data also suggested that C8:0 increased the production of unlabeled AcAc from other unlabeled sources (Figures 1B–D). Additionally, the total amount (AUC) of AcAc C2 produced by the end of the experiment was the same for C8:0 and C10:0 experiments. To understand the relationship between AcAc and βHB, it may be relevant to take HMG into consideration. HMG arises from the combination of a two-carbon molecule (AcCoA) and a four-carbon molecule (AcAc). Yet, no HMG C4 was detected in the medium and no difference in total and unlabeled production of HMG between C8:0 and C10:0 could be measured. Under the assumption that an enzyme should catalyze a reaction (in terms of rate) unreceptively of the labeling counterparts, these results indicate possible cellular compartmentalization of ketone bodies production.

Butyrate is downstream of 3-OH-C6:0, and in terms of carbon flow from C8:0-C10:0 to βHB in astrocytes, it can be considered as a “carbon sink” (Figure 2). While significant labeled amount was measured in the medium, unlabeled butyrate was not present, suggesting that the production of butyrate is specific to the labeled substrates. Astrocytes treated with C8:0 secreted more butyrate C4 as compared to C10:0, in line with larger release and intracellular FE of fully labeled 3-OH-C6:0. The production of butyrate C2 was, on the other hand, larger in the case of C10:0 than C8:0, and could arise from scrambling of AcCoA C2 with unlabeled 3-OH-C6:0. The fact that no C2-labeled MCFA and no unlabeled butyrate was detected indicate different production rate and possible cellular compartmentalization. While it is recognized that MCFA undergo β-oxidation in mitochondria, very long chain fatty acids (of length of 24 or higher) are oxidized in peroxisomes with hexanoyl as a final product (Wanders et al., 2001). It was moreover reported that MCFA can be synthetize both in the cytosol by the fatty acid synthase complex (Volpe and Kishimoto, 1972; Angeles and Hudkins, 2016) (KEGG database) and in mitochondria (reversal of β-oxidation) (KEGG database) (Hinsch et al., 1976; Murad and Kishimoto, 1978).

In the description of the FE curves, we included a dilution flux at the level of AcCoA, which not only reflects the production of unlabeled ketone bodies, but is also necessary to explain the important contribution of unlabeled carbons in the FE curves of the MCFA intermediates. The elongation fluxes (from AcCoA to MCFA production) were included based on the mass isotopomer contribution of unlabeled carbons and on the detection of labeled C10:0 and 3-OH-C10:0 in the medium of C8:0-incubated astrocytes (Figure 4). However, the latter was not included in the C8:0 experiment, because intracellular 3-OH-C10:0 and C10:0 were not detectable and associated with high uncertainty, respectively, and therefore could not be fitted by the model.

Analysis of the fluxes obtained with the mathematical model indicated opposite direction of metabolic flux at specific steps (i.e., F_6_-F_6_^inv^ and F_8_-F_8_^inv^), suggesting different C8:0 and C10:0 β-oxidation pathways. In particular in the C8:0 experiment, F_6_-F_6_^inv^ and F_8_-F_8_^inv^ are directed toward the production of βHB consistent with larger production of βHB C4 and AcAc C4. In the case of C10:0, unlike C8:0, the fraction of labeled carbons drastically decreased at the level of C8:0 (about 5% at 360 min) and stayed stable thereafter (i.e., in 3-OH-C8:0 and 3-OH-C6:0), suggesting that an important carbon flow from unlabeled sources (F_13_) at the level of AcCoA must occur and contribute to the upstream pools (notably through F_8_^inv^ and F_6_^inv^). As a result, even if both C8:0- and C10:0-treated astrocytes rely on significant contribution of unlabeled substrates, a larger dilution occurs in the case of C10:0. Amaral et al. calculated that the consumption rate of isoleucine and leucine are about 10 times smaller than for glucose (Amaral et al., 2011), in line with the general observation that glucose is the main energy substrate for the brain cells including astrocytes (Zwingmann and Leibfritz, 2003; Thevenet et al., 2016). Therefore, it seems reasonable to attribute the dilution at the level of AcCoA (F_13_) to glucose. In fact, as reported in our previous study (Thevenet et al., 2016), the large lactate/βHB ratio secretion rate and the unchanged ATP levels when inhibiting mitochondrial ATP synthesis indicate that glucose remains the main energy substrate in our experimental conditions even in the presence of MCFA. Interestingly, reduced glycolysis and decrease phosphofructokinase activity in the case of C8:0 was reported by Tan et al. (2017), and larger lactate release upon C10:0 as compared to C8:0 was measured by Thevenet et al. (2016). F_13_ was estimated to be 0.187 ± 0.017 μmol/g prot/min and 0.490 ± 0.045 μmol/g prot/min in the C8:0 and C10:0 experiment, respectively, which is in line with these previous reports (Thevenet et al., 2016; Tan et al., 2017).

While the secretion of ketone bodies comprised mostly of the unlabeled fractions, larger amounts of labeled than unlabeled MCFA intermediates were secreted (Figure 4). Moreover, the extracellular secretion profile of the MCFA intermediates was different in the C8:0 and C10:0 experiments. In the case of C8:0, labels were mostly secreted as hydroxy-(3-OH) molecules, except for 3-OH-C10:0 that was secreted in a larger extent in the C10:0 condition. The opposite direction of metabolic flux estimated in the present study suggests different C8:0 and C10:0 β-oxidation pathways, and potential modulation when both MCFA are simultaneously provided (Khabbush et al., 2017). These observations support our hypothesis that C8:0 and C10:0 could exhibit different biological functions due to their different metabolism, and that their beneficial effects may not be limited to production of ketones. Interestingly, 3-OH-C10:0 was shown to depolarize the squid giant axon in inhibiting the sodium channels (Takenaka et al., 1981) and C10:0 was reported to be more anticonvulsant than C8:0 (Chang et al., 2016; Tan et al., 2017). In addition to the direct inhibition of the α-amino-3-hydroxy-5-methyl-4-isoxazolepropionic acid (AMPA) receptors (Chang et al., 2016), we speculate that this additional anti-seizure effect could be due to production of 3-OH-C10:0; however, this requires further investigation. We, moreover, observed that secretion of C6:0 was higher in C10:0 as compared to C8:0. Although the function of C6:0 is unclear, it is worth mentioning that C6:0 was shown to contribute to energy metabolism (Ishiwata et al., 1996), as well as to induce neurite growth (Kamata et al., 2007). All together, these results point to different metabolic network activation and biological functions depending on the carbon length of the substrate. This suggests that the underlying enzymes are substrate-specific, preferentially catalyzing molecules of specific chain lengths and/or adapting to the requirements of specific cellular states, potentially impacting differently the cellular environment.

Finally, an increase in intracellular unlabeled glutathione concentration was observed over time. This increase was significantly higher by the end of the experiment in the C10:0 condition, indicating potential benefits of C10:0 as compared to C8:0 in terms of antioxidant properties. This observation is consistent with the earlier findings of increased brain reduced glutathione levels in rats given C10:0-enriched oil (Sengupa and Ghosh, 2012) and higher expression of genes regulating the expression of antioxidant enzymes in the hippocampus of mice fed with C10:0 as compared to C8:0 (Tan et al., 2017), suggesting therefore potential beneficial effects related to C10:0. Although further experiments are needed to further assess the link between glutathione and C10:0, it is noteworthy that C10:0 is an agonist of the peroxisome proliferator-activated receptor (PPAR) α (Malapaka et al., 2012) involved in glutathione level modulation (Abdelmegeed et al., 2009).

In conclusion, while C8:0 and C10:0 appear structurally similar, as they differ in length by only two carbons, the metabolic pathways underlying their metabolism are different in human iPSC astrocytes. In particular, flux analysis indicate opposite direction of metabolic flux at specific steps, with an important fraction of unlabeled sources contributing to the elongation steps in the case of C10:0, suggesting different C8:0 and C10:0 β-oxidation pathways. The total extracellular concentrations and secretion rates of βHB, AcAc and butyrate are higher and faster, respectively, in the case of C8:0, while intracellular glutathione formation and secretions of 3-OH-C10:0 and C6:0 were larger with C10:0. Overall, the findings suggest MCFA-specific ketometabolic traits, which could be of interest for personalized nutritional intervention and the design of optimal ketogenic diet supplementation. Further studies involving different ratios of C8:0/C10:0 in appropriate models of glucose metabolism impairment are required to investigate sustainable and targeted ketone production, with optimal antioxidant and anticonvulsant impacts.

## Data Availability

All datasets generated for this study are included in the manuscript and/or the Supplementary Files.

## Author Contributions

MM and SS designed the study. AC performed the biochemical pathway analysis *in silico*. JT performed the cell experiment. NC performed the LC-MS measurements. SS analyzed the data and wrote the manuscript. MM, SS, and AC interpreted the data. MM, AW, NC, and AC revised the manuscript.

### Conflict of Interest Statement

All authors of this study are employees of Nestlé.

## Abbreviations

AcAc: Acetoacetate
AcCoA: Acetyl-CoA
ACP: Acyl-carrier protein
AMPA: α-Amino-3-hydroxy-5-methyl-4-isoxazolepropionic acid
AUC: Area under the curve
βHB: β-Hydroxybutyrate
FE: Fractional enrichment
HMG: 3-Hydroxy-3-methyl glutarate
iPSC: Induced pluripotent stem cell-derived
LC-MS: Liquid-chromatography mass spectrometry
MC: Monte Carlo
MCFA: Medium-chain fatty acid
MCT: Medium-chain triglyceride
MS: Mass spectrometry
TBI: Traumatic brain injury.
