# Supplementary material for: Differential Metabolism of Medium-Chain Fatty Acids in Differentiated Human-Induced Pluripotent Stem Cell-Derived Astrocytes
Source: Front Physiol. 2019 Jun 4;10:657. doi: 10.3389/fphys.2019.00657 (PMC6558201; doi:10.3389/fphys.2019.00657)
Supplement: Supplementary file 1 [file Data_Sheet_1.pdf]

Supplementary Material

1 Supplementary Data

Mass isotopomer-balance equations for C8:0 :

$$\frac{d}{dt}\begin{bmatrix} C8{:}\,0_0 \\ C8{:}\,0_8 \end{bmatrix} = \frac{1}{C8{:}\,0}\left(F_5\begin{bmatrix} C8{:}\,0_0^{medium} \\ C8{:}\,0_8^{medium} \end{bmatrix} - F_5^{inv}\begin{bmatrix} C8{:}\,0_0 \\ C8{:}\,0_8 \end{bmatrix} - F_6\begin{bmatrix} C8{:}\,0_0 \\ C8{:}\,0_8 \end{bmatrix} + F_6^{inv}\begin{bmatrix} 3OHC8{:}\,0_0 \\ 3OHC8{:}\,0_8 \end{bmatrix}\right)$$

$$\frac{d}{dt}\begin{bmatrix} 3OHC8{:}\,0_0 \\ 3OHC8{:}\,0_8 \end{bmatrix} = \frac{1}{3OHC8{:}\,0}\left(F_6\begin{bmatrix} C8{:}\,0_0 \\ C8{:}\,0_8 \end{bmatrix} - F_6^{inv}\begin{bmatrix} 3OHC8{:}\,0_0 \\ 3OHC8{:}\,0_8 \end{bmatrix} - F_8\begin{bmatrix} 3OHC8{:}\,0_0 \\ 3OHC8{:}\,0_8 \end{bmatrix} + F_8^{inv}\begin{bmatrix} C6{:}\,0_0 \cdot AcCoA_0 \\ C6{:}\,0_6 \cdot AcCoA_2 \end{bmatrix} - F_7\begin{bmatrix} 3OHC8{:}\,0_0 \\ 3OHC8{:}\,0_8 \end{bmatrix}\right)$$

$$\frac{d}{dt}\begin{bmatrix} C6{:}\,0_0 \\ C6{:}\,0_6 \end{bmatrix} = \frac{1}{C6{:}\,0}\left(F_8\begin{bmatrix} 3OHC8{:}\,0_0 \\ 3OHC8{:}\,0_8 \end{bmatrix} - F_8^{inv}\begin{bmatrix} C6{:}\,0_0 \\ C6{:}\,0_6 \end{bmatrix} - F_{10}\begin{bmatrix} C6{:}\,0_0 \\ C6{:}\,0_6 \end{bmatrix} + F_{10}^{inv}\begin{bmatrix} 3OHC6{:}\,0_0 \\ 3OHC6{:}\,0_6 \end{bmatrix} - F_9\begin{bmatrix} C6{:}\,0_0 \\ C6{:}\,0_6 \end{bmatrix}\right)$$

$$\frac{d}{dt}\begin{bmatrix} 3OHC6{:}\,0_0 \\ 3OHC6{:}\,0_6 \end{bmatrix} = \frac{1}{3OHC6{:}\,0}\left(F_{10}\begin{bmatrix} C6{:}\,0_0 \\ C6{:}\,0_6 \end{bmatrix} - F_{10}^{inv}\begin{bmatrix} 3OHC6{:}\,0_0 \\ 3OHC6{:}\,0_6 \end{bmatrix} - F_{12}\begin{bmatrix} 3OHC6{:}\,0_0 \\ 3OHC6{:}\,0_6 \end{bmatrix} + F_{12}^{inv}\begin{bmatrix} AcCoA_0 \cdot AcCoA_0 \cdot AcCoA_0 \\ AcCoA_2 \cdot AcCoA_2 \cdot AcCoA_2 \end{bmatrix} - F_{11}\begin{bmatrix} 3OHC6{:}\,0_0 \\ 3OHC6{:}\,0_6 \end{bmatrix}\right)$$

$$\frac{d}{dt}\begin{bmatrix} AcCoA_0 \\ AcCoA_2 \end{bmatrix} = \frac{1}{AcCoA}\left(F_8\begin{bmatrix} 3OHC8{:}\,0_0 \\ 3OHC8{:}\,0_8 \end{bmatrix} - F_8^{inv}\begin{bmatrix} AcCoA_0 \\ AcCoA_2 \end{bmatrix} + F_{12}\begin{bmatrix} 3OHC6{:}\,0_0 \\ 3OHC6{:}\,0_6 \end{bmatrix} - F_{12}^{inv}\begin{bmatrix} AcCoA_0 \cdot AcCoA_0 \cdot AcCoA_0 \\ AcCoA_2 \cdot AcCoA_2 \cdot AcCoA_2 \end{bmatrix} - F_{14}\begin{bmatrix} AcCoA_0 \cdot AcCoA_0 \\ AcCoA_2 \cdot AcCoA_2 \end{bmatrix} + F_{13}\begin{bmatrix} 1 \\ 0 \end{bmatrix}\right)$$

Mass isotopomer-balance equations for C10:0:

$$\frac{d}{dt}\begin{bmatrix} C10{:}\,0_0 \\ C10{:}\,0_{10} \end{bmatrix} = \frac{1}{C10{:}\,0}\left(F_1\begin{bmatrix} C10{:}\,0_0^{medium} \\ C10{:}\,0_{10}^{medium} \end{bmatrix} - F_1^{inv}\begin{bmatrix} C10{:}\,0_0 \\ C10{:}\,0_{10} \end{bmatrix} - F_2\begin{bmatrix} C10{:}\,0_0 \\ C10{:}\,0_{10} \end{bmatrix} + F_2^{inv}\begin{bmatrix} 3OHC10{:}\,0_0 \\ 3OHC10{:}\,0_{10} \end{bmatrix}\right)$$

$$\frac{d}{dt}\begin{bmatrix} 3OHC10{:}\,0_0 \\ 3OHC10{:}\,0_{10} \end{bmatrix} = \frac{1}{3OHC10{:}\,0}\left(F_2\begin{bmatrix} C10{:}\,0_0 \\ C10{:}\,0_{10} \end{bmatrix} - F_2^{inv}\begin{bmatrix} 3OHC10{:}\,0_0 \\ 3OHC10{:}\,0_{10} \end{bmatrix} - F_4\begin{bmatrix} 3OHC10{:}\,0_0 \\ 3OHC10{:}\,0_{10} \end{bmatrix} + F_4^{inv}\begin{bmatrix} C8{:}\,0_0 \cdot AcCoA_0 \\ C8{:}\,0_8 \cdot AcCoA_2 \end{bmatrix} - F_3\begin{bmatrix} 3OHC10{:}\,0_0 \\ 3OHC10{:}\,0_{10} \end{bmatrix}\right)$$

$$\frac{d}{dt}\begin{bmatrix} C8{:}\,0_0 \\ C8{:}\,0_8 \end{bmatrix} = \frac{1}{C8{:}\,0}\left(F_4\begin{bmatrix} 3OHC10{:}\,0_0 \\ 3OHC10{:}\,0_{10} \end{bmatrix} - F_4^{inv}\begin{bmatrix} C8{:}\,0_0 \\ C8{:}\,0_8 \end{bmatrix} - F_6\begin{bmatrix} C8{:}\,0_0 \\ C8{:}\,0_8 \end{bmatrix} + F_6^{inv}\begin{bmatrix} 3OHC8{:}\,0_0 \\ 3OHC8{:}\,0_8 \end{bmatrix} - F_5^{inv}\begin{bmatrix} C8{:}\,0_0 \\ C8{:}\,0_8 \end{bmatrix}\right)$$

$$\frac{d}{dt}\begin{bmatrix} 3OHC8{:}\,0_0 \\ 3OHC8{:}\,0_8 \end{bmatrix} = \frac{1}{3OHC8{:}\,0}\left(F_6\begin{bmatrix} C8{:}\,0_0 \\ C8{:}\,0_8 \end{bmatrix} - F_6^{inv}\begin{bmatrix} 3OHC8{:}\,0_0 \\ 3OHC8{:}\,0_8 \end{bmatrix} - F_8\begin{bmatrix} 3OHC8{:}\,0_0 \\ 3OHC8{:}\,0_8 \end{bmatrix} + F_8^{inv}\begin{bmatrix} C6{:}\,0_0 \cdot AcCoA_0 \\ C6{:}\,0_6 \cdot AcCoA_2 \end{bmatrix} - F_7\begin{bmatrix} 3OHC8{:}\,0_0 \\ 3OHC8{:}\,0_8 \end{bmatrix}\right)$$

$$\frac{d}{dt}\begin{bmatrix} C6{:}\,0_0 \\ C6{:}\,0_6 \end{bmatrix} = \frac{1}{C6{:}\,0}\left(F_8\begin{bmatrix} 3OHC8{:}\,0_0 \\ 3OHC8{:}\,0_8 \end{bmatrix} - F_8^{inv}\begin{bmatrix} C6{:}\,0_0 \\ C6{:}\,0_6 \end{bmatrix} - F_{10}\begin{bmatrix} C6{:}\,0_0 \\ C6{:}\,0_6 \end{bmatrix} + F_{10}^{inv}\begin{bmatrix} 3OHC6{:}\,0_0 \\ 3OHC6{:}\,0_6 \end{bmatrix} - F_9\begin{bmatrix} C6{:}\,0_0 \\ C6{:}\,0_6 \end{bmatrix}\right)$$

$$\frac{d}{dt}\begin{bmatrix} 3OHC6{:}\,0_0 \\ 3OHC6{:}\,0_6 \end{bmatrix} = \frac{1}{3OHC6{:}\,0}\left(F_{10}\begin{bmatrix} C6{:}\,0_0 \\ C6{:}\,0_6 \end{bmatrix} - F_{10}^{inv}\begin{bmatrix} 3OHC6{:}\,0_0 \\ 3OHC6{:}\,0_6 \end{bmatrix} - F_{12}\begin{bmatrix} 3OHC6{:}\,0_0 \\ 3OHC6{:}\,0_6 \end{bmatrix} + F_{12}^{inv}\begin{bmatrix} AcCoA_0 \cdot AcCoA_0 \cdot AcCoA_0 \\ AcCoA_2 \cdot AcCoA_2 \cdot AcCoA_2 \end{bmatrix} - F_{11}\begin{bmatrix} 3OHC6{:}\,0_0 \\ 3OHC6{:}\,0_6 \end{bmatrix}\right)$$

$$\frac{d}{dt}\begin{bmatrix}AcCoA_0\\AcCoA_2\end{bmatrix} = \frac{1}{AcCoA}\left(F_4\begin{bmatrix}3OHC10:0_0\\3OHC10:0_{10}\end{bmatrix} - F_4^{inv}\begin{bmatrix}AcCoA_0\\AcCoA_2\end{bmatrix} + F_8\begin{bmatrix}3OHC8:0_0\\3OHC8:0_8\end{bmatrix} - F_8^{inv}\begin{bmatrix}AcCoA_0\\AcCoA_2\end{bmatrix} + F_{12}\begin{bmatrix}3OHC6:0_0\\3OHC6:0_6\end{bmatrix} - F_{12}^{inv}\begin{bmatrix}AcCoA_0\cdot AcCoA_0\cdot AcCoA_0\\AcCoA_2\cdot AcCoA_2\cdot AcCoA_2\end{bmatrix} - F_{14}\begin{bmatrix}AcCoA_0\cdot AcCoA_0\\AcCoA_2\cdot AcCoA_2\end{bmatrix} + F_{13}\begin{bmatrix}1\\0\end{bmatrix}\right)$$

2 Supplementary Table

	C8:0		C10:0	
	flux	SD	flux	SD
F_1	NA	NA	0.217	0.069
F_1^{inv}	NA	NA	0.236	0.077
F_2	NA	NA	0.012	0.004
F_2^{inv}	NA	NA	0.027	0.010
F_4	NA	NA	0.020	0.014
F_4^{inv}	NA	NA	0.045	0.015
F_5	0.135	0.029	NA	NA
F_5^{inv}	0.080	0.031	NA	NA
F_6	0.098	0.014	0.091	0.131
F_6^{inv}	0.043	0.011	0.238	0.129**
F_8	0.140	0.030	0.000	0.000***
F_8^{inv}	0.109	0.027	0.221	0.074**
F_{10}	0.532	0.379	0.000	0.000**
F_{10}^{inv}	0.877	0.452	0.241	0.057**
F_{12}	0.000	0.000	0.708	0.666*
F_{12}^{inv}	0.147	0.031	0.930	0.644**
F_{13}	0.187	0.017	0.490	0.045***
Calculated differences				
$F_6 - F_6^{inv}$	0.056	0.018	-0.147	0.184**
$F_8 - F_8^{inv}$	0.031	0.041	-0.221	0.074*
$F_{10} - F_{10}^{inv}$	-0.345	0.590	-0.241	0.057
$F_{12} - F_{12}^{inv}$	-0.147	0.031	-0.222	0.926

Table S1 : Fluxes (in µmol/g prot/min) resulting from the best fit of the mathematical model of β-oxidation. NA : non-available. * $p < 0.05$; ** $p < 0.01$; ***$p < 0.001$

3 Supplementary Figure

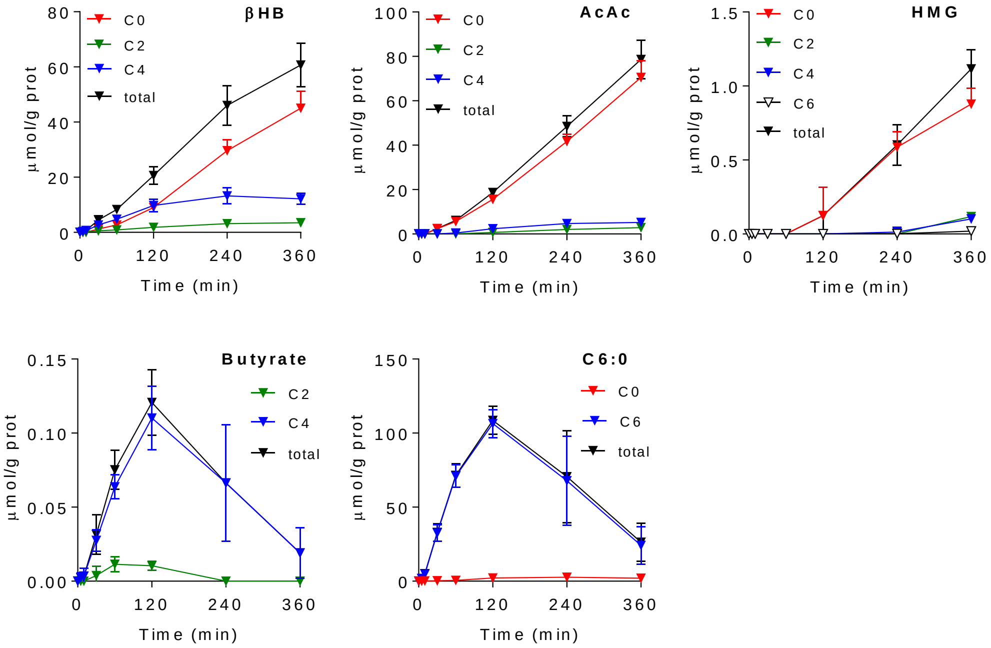

Figure S1 : Secretion rates of selected ketone bodies, HMG, butyrate and C6:0 determined from the total extracellular concentrations experimentally measured in HepG2 cells incubated with [U-^{13}C]-C8:0 (mean±SD, n=3). Secretion of βHB and AcAc was detected at time 5 min and 30 min, respectively, consistent with the general observation that the liver is the major organ for ketone production (Evans et al., 2017). Secretion of HMG, butyrate and C6:0 was detected at 120 min, 5 min and 5 min, respectively. Detection of mass isotopomers for HMG and butyrate was different as compared to iPSC astrocytes. Concentrations of butyrate and C6:0 peaked at 120 min and then decreased (inverted U-shape). All together, these data show different metabolite quantification in different cell types under similar conditions, supporting the sensitivity and the specificity of the measurements.
